# Smart Bluetooth Stakes: Deployment of Soil Moisture Sensors with Rotating High-Gain Antenna Receiver on Center Pivot Irrigation Boom in a Commercial Wheat Field

**DOI:** 10.3390/s25175537

**Published:** 2025-09-05

**Authors:** Samuel Craven, Austin Bee, Blake Sanders, Eliza Hammari, Cooper Bond, Ruth Kerry, Neil Hansen, Brian A. Mazzeo

**Affiliations:** 1 Department of Electrical and Computer Engineering, Brigham Young University, Provo, UT 84602, USA; 2 Department of Geography, Brigham Young University, Provo, UT 84602, USA; 3 Department of Plant and Wildlife Sciences, Brigham Young University, Provo, UT 84602, USA

**Keywords:** soil moisture, precision agriculture, IoT, Bluetooth Low Energy, sensor networks, water conservation, solar energy harvesting, environmental monitoring

## Abstract

Realization of the goals of precision agriculture is dependent on prescribing irrigation strategies matched to spatiotemporal variations in soil moisture on commercial farms. However, the scale at which these variations occur is not well understood. A high-spatial-density network of sensors with the ability to measure and report data over the course of a growing season is needed. In this work, design of the low-profile Smart Bluetooth Stake spatiotemporal soil moisture mapping system is presented. Smart stakes use Bluetooth Low Energy to communicate 64 MHz soil moisture impedance measurements from ground level to a receiver mounted on the center-pivot irrigation boom and equipped with a rotating high-gain parabolic antenna. Smart stakes can remain in the ground throughout the entire growing season without disrupting farm operations. A system of 86 sensors was deployed on a 50-hectare commercial field near Elberta, Utah, during the final growth stage of a crop of winter wheat. Different receiver antenna configurations were tested over the course of several weeks which included two full irrigation cycles. In the high-gain antenna configuration, data was successfully collected from 75 sensors, with successful packet transmission at ranges of approximately 600 m. Enough data was collected to construct a spatiotemporal moisture map of the field over the course of an irrigation cycle. Smart Bluetooth Stakes constitute an important advance in the spatial density achievable with direct sensors for precision agriculture.

## 1. Introduction

Agricultural use accounts for 70% of freshwater withdrawals worldwide [1,2] and up to a third of that use outstrips sustainable supply [3,4]. An urgent need exists to improve Water Use Efficiency (WUE) in irrigation to safeguard future water and food supplies. Precision irrigation seeks to significantly improve WUE by using sensed data and modeling to make intelligent decisions about where, how much, and when to irrigate.

Variations in water needs occur not just spatially over a field, but temporally as well: even within the same growing season, the behavior of different moisture zones can dramatically change [5]. Understanding these needs motivates creation of custom watering schemes that allocate as much water as is needed and thereby avoid both under- and over-watering. It has been repeatedly demonstrated that such strategies can reduce wasted irrigation water without sacrificing crop yields [6,7,8] and provide meaningful economic benefits as well as environmental ones [9,10], and yet adoption has remained low. Many farmers still apply the same water prescriptions uniformly across their fields. In 2017, 75% of farmers in the United States used rule-of-thumb methods to schedule irrigation rather than methods informed by sensors, and that number had not changed as of 2023 [11].

A key barrier to the adoption of data-driven approaches is the collection of data at necessary spatial and time scales [12]. Because spatial variations in soil moisture behavior occur at scales as small as tens of meters [13] and evolve over time [14], a viable sensing system must be capable of monitoring soil moisture at sufficiently high spatial resolution. Significant effort has gone into overcoming the inherent time and spatial resolution constraints of remote sensing approaches, but to date the best of these struggle to achieve shorter measurement intervals than 24 h [15], which may not be sufficient to attain optimal results [16]. Direct sensing methods are already capable of performing measurements in real time, and if barriers to high-spatial-density deployments can be overcome, they present a promising path forward to improving the accessibility of precision irrigation.

Although many different Wireless Sensor Networks (WSNs) for direct soil moisture sensing in precision agriculture have been demonstrated [17], their adoption remains low, reflecting the lack of a system practical enough for widespread use. This is likely because of the many separate but interdependent design constraints necessary for a successful system: low cost per sensor, ease of deployment, unintrusiveness to farm operations, self-contained power, and the ability to transmit measurements for real-time use.

All of these barriers have been successfully overcome by different systems but, to date, no system has fulfilled all the requirements at once. Systems like [18,19] have demonstrated transmission from multiple sensors across a commercial-size field, but with cost per sensor above USD 500. Some systems such as [20,21] require cables to be run between nodes and hubs, which would obstruct farm equipment. Several networks achieved low cost per sensor and good transmission range, but deployment in high numbers at long distances was never considered or attempted [22,23,24,25]. To our knowledge, no system of low-cost wireless direct soil moisture sensors with more than 24 nodes has been deployed in a commercial-sized field.

Various sub-GHz transmission protocols are natural candidates for the type of system proposed, with several advantages over higher-frequency protocols like Bluetooth Low Energy (BLE). Lower frequency transmissions are inherently less susceptible to attenuation from obstacles such as crop canopies, and various protocols (e.g., DASH7, LoRa, Sigfox, etc.) can be configured for receive sensitivity as low as −140 dBm, compared to the max receive sensitivity of the smart stake receiver (before adding antenna gain) of −106.4 dBm. However, these protocols have downsides that limit their effectiveness for high-density, low-cost sensor networks and make the applicability of BLE clearer.

LoRa is a relevant example representative of several other possible protocols. The high receive sensitivity of LoRa comes at the cost of significantly longer time over air (ToA) for each broadcast: transmissions of several dozen bytes in the highest range configuration can take longer than 1500 ms, compared to a 125 kbps GAP-Coded PHY BLE broadcast packet with a 253-byte payload which takes about 4.5 ms [26]. Both systems must consume close to their peak broadcasting power throughout transmission, so if a LoRa broadcast takes 300 times longer it will also consume about 300 times as much energy. This additional energy consumption is not insurmountable, but it does require larger and more costly energy harvesting and storage systems as compared to the BLE equivalent.

The long ToA of LoRa broadcasts also requires careful consideration of throughput for the proposed network topology, as asynchronous one-way broadcasts will inevitably have packet collisions. In [27], it was shown that with a requirement for a 90% packet delivery rate and a broadcast interval of 16.7 min and with 20-byte payloads, a single LoRa network could not scale past 120 nodes due to collisions. Shortening broadcast intervals, increasing spreading factor for higher range, and increasing payload length will all significantly worsen this problem.

The much shorter broadcast time for BLE means that hundreds of individual sensors can successfully advertise to a single gateway even if the broadcast interval is as short as 5 s [28]. With 5 min broadcast intervals, the Smart Bluetooth Stake system could support thousands of individual nodes, if needed. Collecting a high number of packets is desirable (and a design goal of the Smart Bluetooth Stake system) because attenuation and resulting sensitivity to crop canopy parameters can also be considered a feature of using the 2.4 GHz band to potentially infer crop characteristics [29].

Transmissions at 933 MHz have a 32 cm wavelength compared to the 2.4 GHz 12.5 cm wavelength. Lower frequency antennas must be either larger, less efficient, or of lower gain and angular isolation (directivity) than comparable 2.4 GHz antennas. Because antennas with such high directivity are not as readily available for frequencies lower than 2.4 GHz, these other protocols are not able to take advantage of pattern diversity available with a rotating high-gain antenna. Pattern diversity reduces the collision rate of signals in high-density networks by creating spatial sectors of prioritized transmitters. Additionally, simple antennas for lower frequencies are generally larger than their 2.4 GHz equivalents or require helical windings or other shapes to meet the quarter-wavelength requirement for many antenna designs. All else being equal, higher frequencies mean smaller packages for transmitters and receivers.

The use of irrigation booms as an available platform for mounting receivers to collect transmitted signals significantly shortens the path length through the high-attenuation crop canopy and is different than other studies in which the transmitters and receivers are at the same height [30]. This is particularly true if only the data in front of the moving boom is needed to make informed precision irrigation decisions in a closed-loop system. Because sensors need to be placed close enough to the ground so that farm equipment can easily pass over them, the requirement for small antennas on the transmitters is a necessity that is more easily achieved with a higher frequency transmission protocol.

LoRa SoCs that combine MCUs and radios are currently more expensive than BLE SoCs by approximately USD 5–10 at scale, which would increase the total sensor cost by as much as 33%. The use of separate low-power MCUs and radios would bring the cost close to parity, but at the cost of increasing implementation complexity.

**Figure 1:**
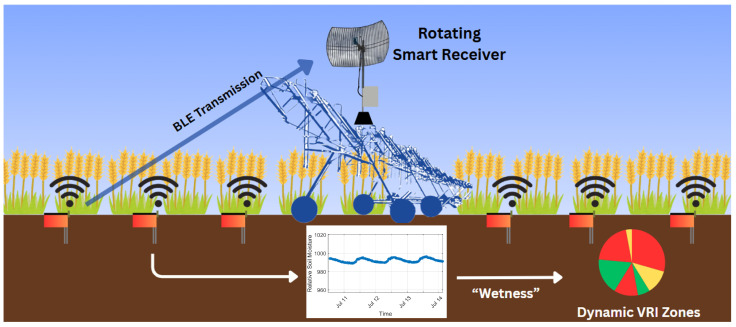
The Smart Bluetooth Stake system design consists of a large number of inexpensive transmitters deployed in a field that send unacknowledged BLE advertising broadcasts with a history of sensor data. A Smart Receiver with a high-gain passive antenna mounted on an irrigation boom collects the data transmissions so that spatiotemporal soil moisture distribution maps can be created.

In this work, it is shown that an approach centered on deploying many low-cost sensors can meet all the design requirements in a way that systems of a few, high-cost sensors cannot. The use of highly affordable BLE microchips helps drive the cost per sensor so low that system reliability can be achieved through excess capacity. With 50 to 100 or more sensors deployed, a useful amount of moisture data can be collected even if a high number of sensors fail to communicate reliably. A 30 h running measurement history is recorded in nonvolatile memory and added to each broadcast, so that if only a single packet every 30 h is successfully transmitted, the full hourly measurement history can be reconstructed. Additionally, the system is built around one-way broadcasts from the sensors to the receiver, which significantly increase range and power efficiency by avoiding the challenges of two-way communication. These design decisions allow the Smart Bluetooth Stake system to achieve a cost to spatial resolution ratio multiple times better than any previously demonstrated system. Figure 1 shows an overview of this radical system approach to high-spatial-density sensor data collection.

## 2. Materials and Methods

### 2.1. Smart Bluetooth Stakes

**Figure 2:**
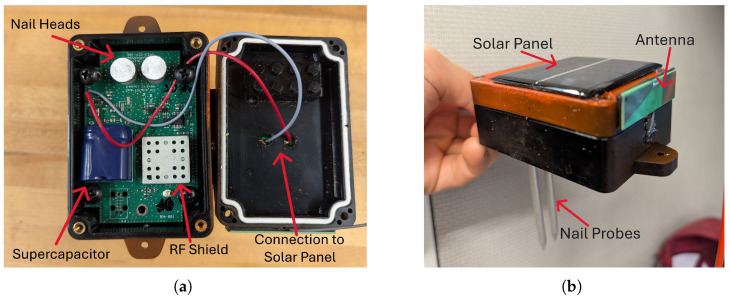
Smart Bluetooth Stake. (**a**) Internal view showing the supercapacitor and nail heads. (**b**) External view with solar panel, nail probes, and antenna.

The design of the individual Smart Bluetooth Stake soil moisture sensor is outlined below, followed by the design of the Smart Receiver data collection station. Figure 2 shows the internal elements and enclosure of a Smart Bluetooth Stake.

#### 2.1.1. Subsystem Power

Though modern low-power IoT microcontroller units (MCUs) can achieve month- or year-long lifetimes with nonrechargeable batteries [31,32], doing so requires careful power management and precludes continuous high-power broadcasts. Using a solar energy harvesting system [33] with a significant power surplus enables Smart Bluetooth Stakes to broadcast regularly at 20 dBm, which significantly boosts range compared to the 0–8 dBm broadcasts typical of low-power IoT devices. It also means Smart Bluetooth Stakes can stay powered in the field indefinitely, even when the majority of sunlight is blocked by the crop canopy.

**Figure 3:**
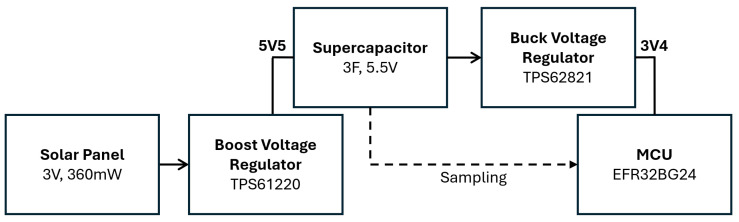
Smart Bluetooth Stake power circuit to boost the solar panel voltage to charge the supercapacitor and then to regulate the voltage to the MCU.

An over-sized solar panel relative to the average energy draw of the Stakes ensures that a power surplus is maintained. As shown in Figure 3 the Smart Bluetooth Stake uses a 3 V, 360 mW solar panel and has an expected average power draw in the hundreds of microwatts. A high-efficiency boost converter that works with input voltages as low as 0.7 V was selected to harvest power from the solar panel even when shade causes the panel to drop below its nominal operating voltage. Energy is stored in a 5.5 V, 3 F supercapacitor. Supercapacitors have superior charge and discharge times, less dependence on temperature, and lower fire risk compared to lithium-ion batteries. Additionally, they do not lose significant capacity over the course of many charge–discharge cycles. However, supercapacitor voltage changes much more as energy is used compared to electrochemical cells, so a high-efficiency buck converter maintains a stable MCU power voltage of 3.4 V as the supercapacitor varies between 3.4 V and 5.5 V.

A resistive divider circuit allows the MCU to measure the supercapacitor voltage. In order to capitalize on spare energy when the supercapacitor has been fully charged, approximately every 6.4 min the system wakes from a low-energy sleeping state and the MCU decides between different broadcast modes depending on the supercapacitor voltage. If the supercapacitor is above 5.3 V, the stake enters high-power broadcasting mode and broadcasts 25 packets per wake. If the supercapacitor is between 3.0 V and 5.3 V, normal broadcasting mode is used and only 10 packets are broadcast per wake. If the supercapacitor falls below 3.0 V the stake enters passive measurement mode, in which no broadcasts are sent, but periodic measurements continue as normal. The stakes can last for several days in this mode because solely recording measurements consumes many times less power than broadcasting: 3.6 millijoules, compared to 70.2 millijoules for a burst of 10 broadcasts. This power management ensures that even if many consecutive hours of rain or other nonideal conditions for energy harvesting prevented the stake from fully recharging, the measurement history would still be recorded. At the time of broadcast, the supercapacitor voltage measurement is sent along with each packet.

#### 2.1.2. Subsystem Nail Probes

**Figure 4:**
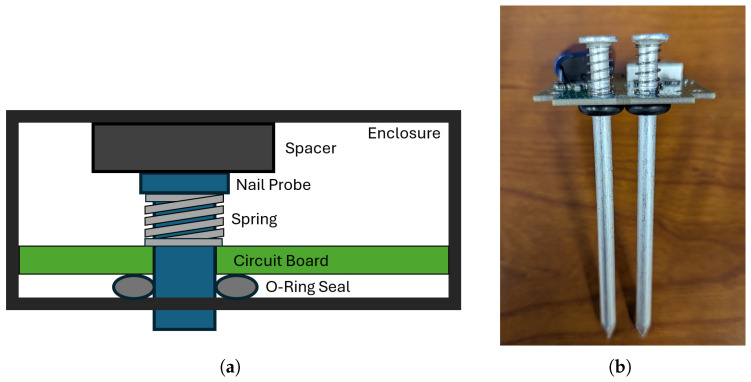
Mechanical implementation of the stake sensor: (**a**) Cross-sectional design of nail probe and circuit board enclosure; (**b**) spring-loaded nail probes and sealing O-rings.

Previous experience has shown that any sensor with probes that penetrate the ground must be structurally rigid so that enough force can be applied to push the probes into hardened, dry soil. Aluminum framing nails (12.7 cm long) are optimized for this role already, are inexpensive, have the required electrical conductivity, and are corrosion-resistant. However, it is also important to prevent force applied to the probes from transferring to the printed circuit board and causing damage if the probes are rigidly attached to the circuit board. To mitigate this problem, as shown in Figure 4, the nails in the Smart Bluetooth Stake are connected to the main circuit board via springs, which maintain reliable electrical contact while absorbing any shock that would otherwise transfer to the circuit board. The springs are sized so that they are partially compressed when the enclosure lid is secured. O-rings and silicone-based plumber’s grease are used underneath the circuit board to waterproof the enclosure. Immersion tests confirmed the arrangement prevented water intrusion.

Prototype tests showed that wireless data transmission performance was better with an exterior antenna, so an Ezurio MAP94045 2.4 GHz PCB trace antenna (Akron, OH, USA) was mounted to the outside of the enclosure with a coaxial connector running to the MCU 2.4 GHz radio transceiver. Even with the additional height of the antenna protruding up to 3 mm, the total stake height above the ground is under 5 cm, which allows regular farm operations, including harvesting, to go on directly over top of the unit.

#### 2.1.3. Subsystem Sensing

**Figure 5:**
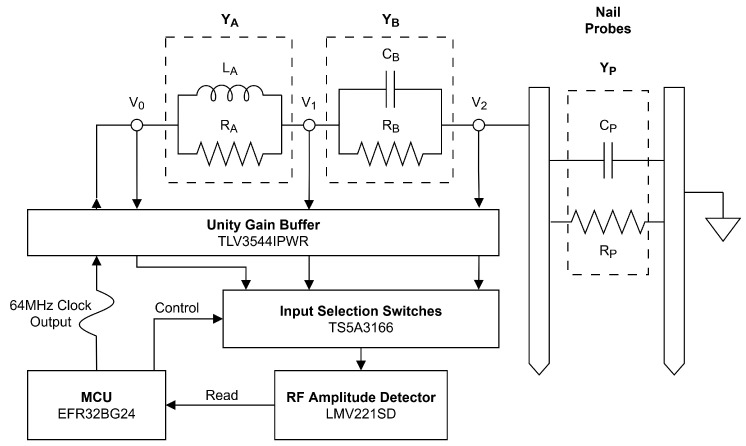
Schematic of moisture measurement circuit using three-voltmeter method to estimate high-frequency complex permittivity of soil between the nail probes.

Low-cost soil moisture sensors typically use either low-frequency capacitive or even DC resistance-based measurements, both of which are prone to accuracy loss due to factors such as changing soil salinity. By estimating the high-frequency complex permittivity of the soil, moisture content and salinity can be separately calculated, leading to better accuracy. Use of the three-voltmeter method to estimate complex permittivity with inexpensive components was demonstrated in [34]. As shown in Figure 5 we implemented this method using the already-available MCU high-frequency clock output at 64 MHz to drive the circuit and employed an RF power measurement IC as an amplitude detector; 64 MHz was chosen for the measurement frequency to increase the strength of the correlation between permittivity and soil moisture [34]. High-frequency operational amplifiers isolate the circuit as waveforms from different sampling points are multiplexed to the RF IC, which then communicates power measurements back to the MCU.

The three-voltmeter method [35] works by creating a voltage divider with three impedance blocks, $Y_{A}$, $Y_{B}$ and $Y_{P}$. The first two consist of circuit elements on the circuit board, each with a known and carefully tuned impedance, and the probe nails make up the third. By measuring the AC amplitude at points $V_{1}$, $V_{2}$, and $V_{3}$, a system of equations is constructed which can be solved for the real and imaginary parts of the complex impedance of the probe. From there, the soil moisture and salinity can be calculated following the method outlined in [34].

The MCU output clock voltage drops significantly if the MCU supply voltage drops below 3.4 V, meaning any measurements taken drop in magnitude correspondingly. The drop is precipitous enough that these points can be easily discarded as outliers, but the result is that accurate measurement histories are not currently recoverable for stakes that spend a long time between 3.4 V and 3.0 V input voltage. For the deployment discussed in this work, the broadcast voltage threshold was deliberately set to 3.0 V in order to maximize the number of broadcasts, as demonstration of successful transmission in the initial deployment was considered more important than preserving full measurement histories. In future, the broadcast voltage threshold will be set to at least 3.5 V to fully preserve measurement histories.

#### 2.1.4. Subsystem Data Storage and Transmission

The required transmission bandwidth for soil moisture measurements is low (on the order of a few tens of bytes per day), so the stakes use BLE GAP-Coded PHY, which trades 8 times lower maximum throughput for an approximately 8 dB increase in RSSI [36]. The broadcasts sent by the stakes are called packets. Because the stakes send unidirectional, unacknowledged broadcasts, no handshaking to coordinate multi-packet data streams is possible, and so the amount of broadcast data is limited to the size of a single advertising packet payload of 253 bytes.

With a simple encoding scheme, each moisture measurement takes up 6 bytes and the packet space is allocated so as to balance measurement period and the extent of measurement history. Prior to this work, both the packet loss rate and the utility of high-time resolution measurements were unknown, and so a middle-of-the-table measurement period of one hour was chosen, allowing for a 30 h history reconstruction. Additionally the most recent hour of measurements at time of broadcast is resolved down to 6.4 min intervals. The data field in each packet is split into two circular buffers, one with 9 spaces to track the last hour of measurements in 6.4 min intervals, and the second with 30 spaces for long-term tracking. Every 60 min, the previous nine 6.4 min interval measurements are averaged and the result stored in the long-term buffer for broadcast. The remaining space in the payload is taken up by header information, including status fields and a counter that is incremented each time the stake takes a measurement, so that the post-processing can resolve the timing of the most recent measurements within the buffers.

Because the stakes do not coordinate their broadcast timing with the receiver, there is risk of significant packet loss from the receiver scanning a different channel at the time when a stake broadcasts to a particular channel. To broadcast at 20 dBm, adaptive channel hopping is required by the FCC Bluetooth specification in order to prevent crowding. For our system, this means that both the channel and arrival time of any given packet are entirely unknown at the time of reception. In order to reduce the chance that a scanner misses a packet because it does not happen to be scanning the correct channel at time of arrival, the stakes are limited to broadcasting on 20 out of the 40 total available channels. In testing this was found to increase packet reception rates by about 50%.

### 2.2. Smart Receivers

**Figure 6:**
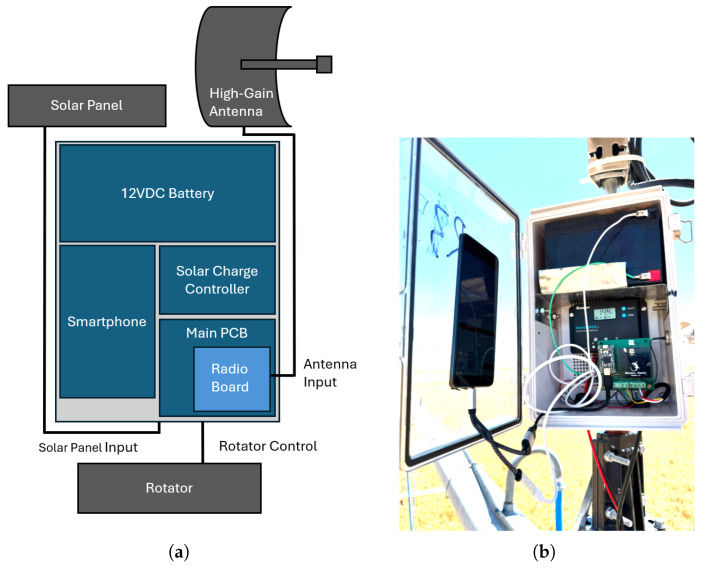
Smart Receiver system. (**a**) Schematic of the main modules. (**b**) Photograph of the assembled Smart Receiver inside the enclosure in the field.

The Smart Receiver is designed to reliably receive BLE packets and upload them to a cloud storage server for real-time access, while being as low-cost as possible. Mounting the receiver on the irrigation boom ensures that as the boom rotates, the receiver gets as close as possible to every point on the field. It also means that the approximately 7 m height of the receiver antenna can mitigate the deleterious transmission effects of placing the stakes at ground level. The hardware layout of the receiver is outlined in Figure 6a and a picture of the main hardware is shown in Figure 6b.

#### 2.2.1. Subsystem Smartphone

Many of the Smart Receiver receiver digital components are consolidated within an inexpensive smartphone. Cellular connectivity, an Inertial Measurement Unit (IMU), a Magnetometer, GPS, control logic, backup battery power, and a touchscreen are all included for a total cost under USD 150.

#### 2.2.2. Subsystem Power

The Smart Receiver station is self-powered to avoid reliance on farm power infrastructure and reduce potential downtime. A 12 Ah, 12 V lead–acid battery provides capacity sufficient for 60 h of continuous operation without any solar charging, and a deliberately over-sized 50 W panel ensures a power surplus even under less-than-ideal conditions. An off-the-shelf consumer-grade solar charge controller provides Maximum Power Point Tracking (MPPT), battery charging, 12 V output for the rotator motor, and 5 V USB output to charge the phone and power the main receiver control board. The average power draw of the system is about 1.5 W.

#### 2.2.3. Subsystem Communication

FCC power transmission limits restrict broadcasting on most channels at any higher than 20 dBm Equivalent Isotropic Radiated Power (EIRP), which would ordinarily curtail the usefulness of a highly directional receiver antenna. Passive reception, on the other hand, can be arbitrarily high-gain without crowding, which means we can use a highly directional 24 dBi parabolic antenna to maximize link budget while maintaining regulatory compliance. Because the system does not use 2-way communication, there is no way for it to synchronize broadcast times and correct for timing drift, so the system assumes no knowledge about broadcast timing. The receiver station therefore continuously rotates so that over time the likelihood of a stake broadcast coinciding with the receiver antenna pointing directly at it is high. A Silicon Labs BGM220S (Austin, TX, USA) radio board is used as a modem and for additional functions described below. The radio board includes an onboard low-gain patch antenna as well as a U.FL output for connecting to the high-gain parabolic antenna. The patch antenna has a more isotropic response which can be used for better omnidirectional reception at the cost of lower maximum range.

#### 2.2.4. Subsystem Rotation and Control

The Smart Receiver uses a Yaesu G-800 (Cypress, CA, USA) high-torque rotator designed for outdoor satellite dish mounting, which is mounted securely to the center-pivot boom using hose clamps and a neoprene sheet to prevent slipping. The rotator contains a potentiometer that returns the current rotation angle as an analog voltage, which is digitized and then used by the phone for control feedback. The receiver rotates across a full clockwise 360° sweep and then full counterclockwise 360° sweep approximately every 56 min. A normal full rotation of the irrigation boom in the field used in this work took about 83.5 h. The potentiometer angle measurements as well as data from the phone’s internal magnetometer are recorded continuously and logged with each received packet (unsynchronized with receiver rotation). The MCU mounted to the radio board controls the antenna rotator via an H-bridge, reads the rotator potentiometer and main 12 V battery voltage, and reports everything back to the phone via a UART to USB bridge. The phone controls the MCU using Silicon Labs’ Network Commander Protocol (NCP) architecture, which allows the MCU to simultaneously report received packets and respond to asynchronous commands from the phone. All of the control logic is implemented on the phone within a fork of a popular open-source USB–serial app.

#### 2.2.5. Subsystem File Storage and Backend

The receiver phone supports multi-generation cellular connectivity (2G–5G) and is equipped with a SIM card and service plan that allows it to periodically upload received packets and diagnostic data to a Google Firebase server. From there, the data are pulled down and parsed using Python 3.13.7. In future, these data can be shared directly into commercial irrigation management APIs for closed-loop control.

### 2.3. Use of AI

Various Large Language Models were used throughout this project, mostly as a prototyping and troubleshooting tool for MATLAB 2024b and Java code and as an aid for quickly generating code for plots during data analysis.

## 3. Field Experiment

**Figure 7:**
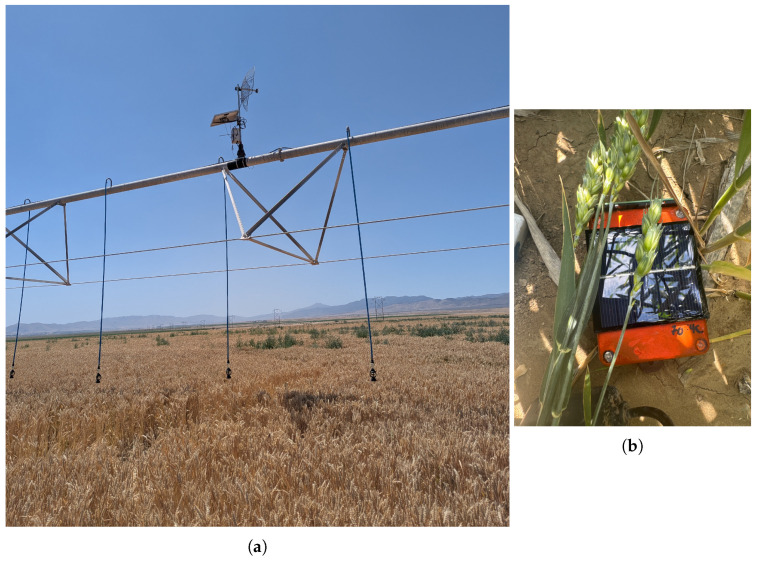
Field deployments near Elberta, Utah. (**a**) Smart Receiver mounted on irrigation boom. (**b**) Smart Bluetooth Stake embedded in soil.

Eighty-six Smart Bluetooth Stakes and one boom-mounted Smart Receiver were deployed to a commercially farmed field in Elberta, Utah, on 15 June 2025, as shown in Figure 7. During the course of the deployment, the irrigation boom completed two full rotations, after which the winter wheat growing in the field was left to dry prior to a target harvest date of 22 July 2025.

### 3.1. Overview of Elberta Field

The field (40°03′02.0″ N, 111°56′25.9″ W), near Elberta, Utah, is irrigated using a center-pivot sprinkler system (Valley Irrigation, Omaha, NE, USA) with a 400 m radius, covering an area of approximately 50 ha. The crop is winter wheat (*Triticum aestivum* L.), planted in October 2024, and was in the grain ripening stage at the time of sensor installation. The dominant soil series is Genola silt loam. Analysis of the surface soil (0–15 cm) showed a composition of sand, silt, and clay fractions of 0.30, 0.42, and 0.28, respectively, along with an organic matter content of 0.26, an alkaline pH of 8.0, and an electrical conductivity of 1.1 dS m^−1^.

### 3.2. Stake Deployment Pattern

**Figure 8:**
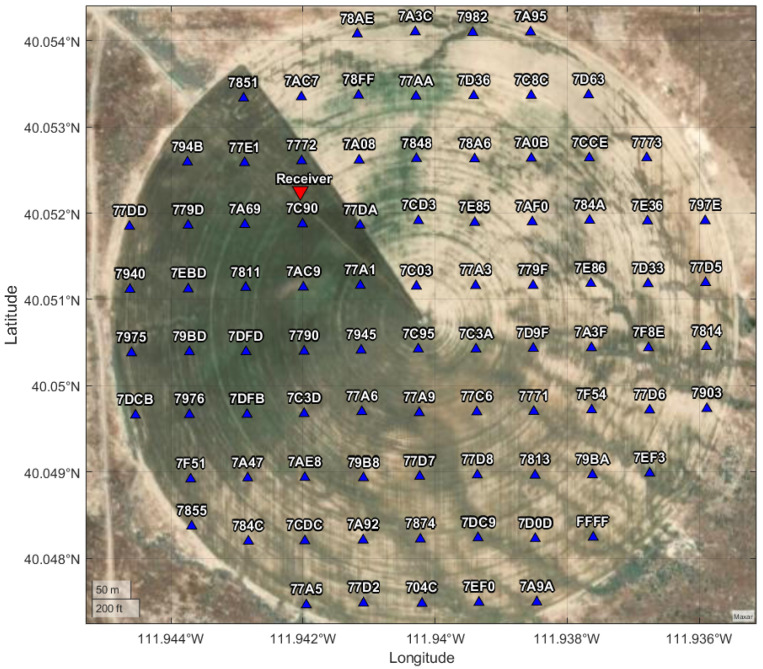
Stake locations as deployed in field, on a 75 m grid with 86 total sensors. Designators correspond to the last 4 characters in each stake’s factory-assigned Universally Unique Identifier (UUID). One stake had its label wiped off and so is designated ‘FFFF’. Base map imagery © Maxar Technologies via MATLAB geoaxes.

Bare soil imagery with a 1 m pixel size from the National Aerial Imagery Program (NAIP) for the field site were downloaded from USGS Earthexplorer. Variograms were computed for different wavebands from the imagery and these had ranges between 240 and 312 m. Following the advice of [37], a suitable grid-spacing interval for this field would be approximately half of the variogram range, i.e., between 120 and 166 m at a minimum. A shape file of the field boundary was used in SpaceStat [38] to generate grids of points with different spacings for first deployment with as many sensors as had been constructed by the time of deployment. A 75 m grid with 86 points in total was used for this deployment. This means that the data were markedly more dense than minimum requirements given the spatial structure of variation observed in the field through aerial imagery. Using more dense data than necessary at this stage allows sub-sampling of the grid to determine if fewer sensors may be needed to properly characterize the spatial variation in soil moisture patterns, and also allows for over-capacity when some sensors fail to send a signal. A Juniper Systems Geode GNS3 GPS unit (Juniper Systems, Logan, UT, USA) was used along with the arcGIS field maps app to place the sensors in the field in the grid locations generated by SpaceStat, as shown in Figure 8.

### 3.3. Deployment Timing and Phases

The sensors were deployed to the field on 15 June 2025 and remained untouched throughout the duration of the field test. However, the boom-mounted Smart Receiver had several configuration changes and fixes over the following weeks. Between 15 and 26 June, a problem in the packet parsing code on the smartphone meant that only about 10% of the received packets were actually processed and uploaded; a fix was applied on 26 June. When the fix was applied, the solar charge controller on the Smart Receiver was left incorrectly configured, so that the battery ran out of charge on the afternoon of 29 June. This was fixed and the boom-mounted receiver was brought back online on 4 July. On 10 July, the Smart Receiver was switched from the low-gain patch antenna to the high-gain parabolic antenna. On 14 July, the phone USB to serial code crashed at 04:58, likely due to the maximum debug log size being exceeded, and was manually reset at 14:05. Throughout the experiment, the boom-mounted receiver remained fixed at the same point on the irrigation boom.

## 4. Results

### 4.1. Packet Reception Rate

**Figure 9:**
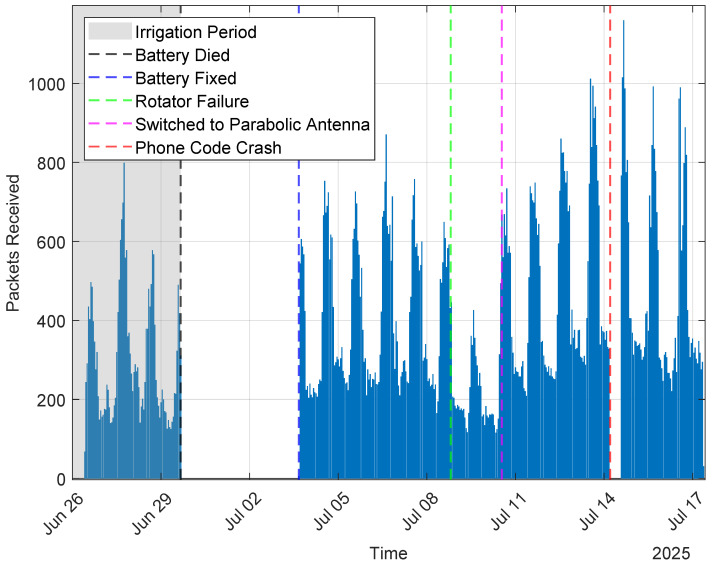
Packets received per hour by boom-mounted receiver. Various fixes and hardware changes demarcated.

Of the 86 stakes deployed as shown in Figure 8, packets from 75 were received in the first 30 days following deployment. The hourly packet reception rates for the boom receiver are shown in Figure 9, along with timestamps to indicate various changes to the receiver that occurred during the recorded period.

**Figure 10:**
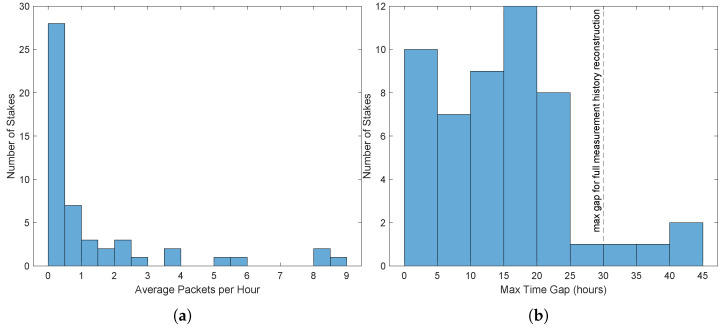
Summary of packet reception performance over 10–14 July 2025, with parabolic antenna and static boom. (**a**) Average number of unique packets received per hour per stake. (**b**) Maximum observed time between any two received packets for each stake.

In order to fully reconstruct the measurement history from a sensor, a packet must be received at least once every 30 h. Figure 10 shows packet reception performance over the course of 4 days during the final drying stages of the winter wheat, when irrigation events had ceased and so the boom did not move. During that period, 51 smart stakes successfully communicated frequently enough for a full measurement history to be reconstructed. The period shown is from after 10 July when the receiver was equipped with a high-gain antenna; prior to 10 July when the boom was actively irrigating, the receiver was using a patch antenna, and had similar performance with the caveat that stakes were only detected when the boom had rotated the receiver to within about 200 m of them. With either antenna, the majority of stakes within the reception range at a given time were detected frequently enough for full measurement history reconstruction.

### 4.2. Stake Power

**Figure 11:**
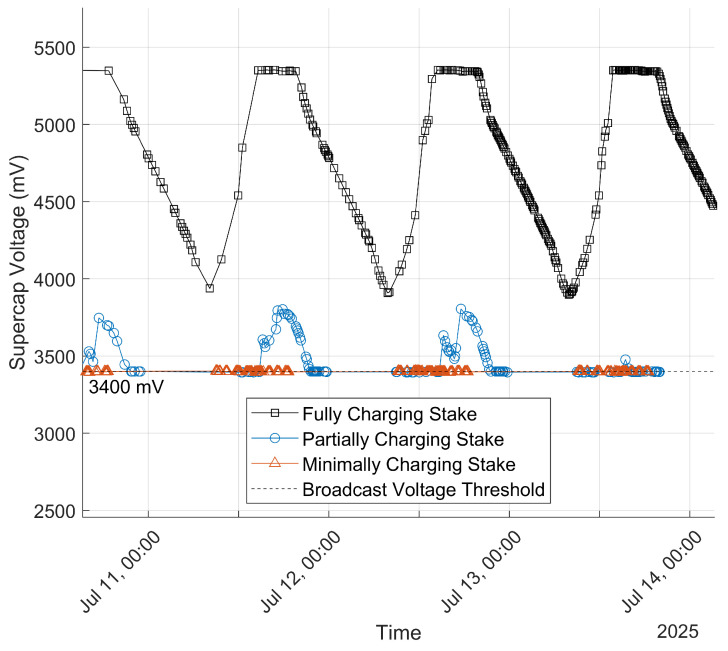
Reported supercapacitor voltages for three different deployed stakes.

The solar energy harvesting on the smart stakes achieved partial success in this test, with only about half of the stakes able to consistently charge to their full capacity. A selection of reported stake supercapacitor voltages is shown in Figure 11. In the ideal case reached by about 50% of the stakes, the supercapacitor voltage never drops below the broadcast threshold, with long periods at the max charge voltage. About 30% showed worse performance where the supercapacitor voltage was able to climb above the threshold but only briefly. The remaining 20%, corresponding to about 14 of the 75 successful stakes, never climbed above the broadcast threshold. Repeated broadcasts at the threshold are evidence of a charging rate slow enough that the 6.4 min sleep period is not long enough for the stake to harvest enough energy to climb past the threshold.

### 4.3. Moisture Estimation

The high-frequency complex soil permittivity measurement approach implemented in the circuit shown in Figure 5 requires careful tuning of the relative magnitudes of the different impedance blocks, including the nail probes, for the desired sensitivity and accuracy to be achieved. The dominant factor in the range of measured impedance values in our system is the distance between the probe nails, and the component values in the design were chosen assuming a consistent 1 cm separation between the nail probes. Unfortunately, higher than anticipated mechanical deviations on the stake enclosures caused significant variation in the probe nail separation between different stakes, from nearly touching to over 2 cm. The achieved sensitivity therefore varied significantly between stakes, preventing accurate moisture estimation for about 30% of the deployed stakes. This also prevented the use of calibrated laboratory measurements against commercial probes that were obtained using reference versions of the stakes.

Given these measurement considerations and the availability of historical soil moisture data for this Elberta field, in this deployment a simplified model of the measurement circuit as a voltage divider is used to estimate moisture, with higher measured values corresponding to a lower proportion of energy dissipated between the probe nails, and therefore lower soil moisture. Previous work in the same field [39] indicates a range of volumetric water content (VWC) between 0.15 and 0.33. The range for each stake was normalized so that the lowest estimated moisture and highest estimated moisture map to the established VWC range. It is clear that historical data may not always be available for commercial fields, but this approach allows for a reasonable soil moisture scaling given the data that was collected.

**Figure 12:**
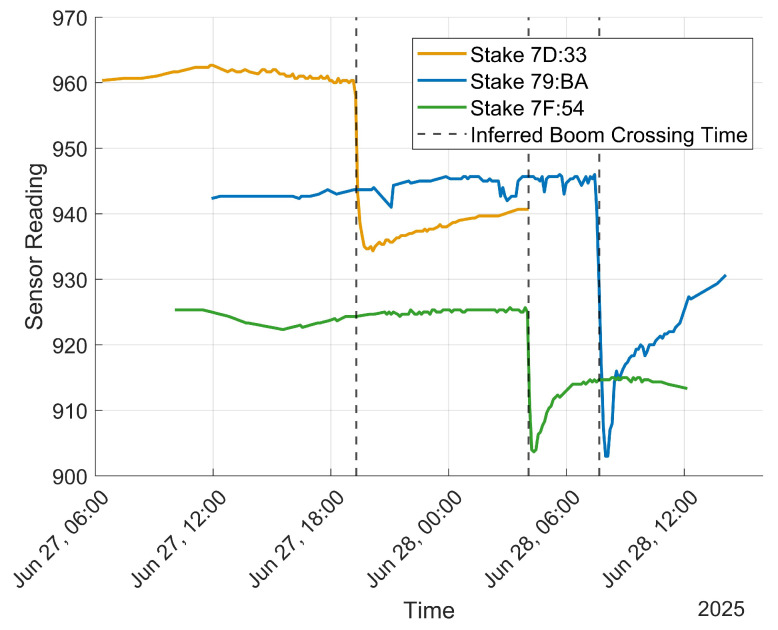
Measurements reported by three stakes at different points in the field, with the inferred time the irrigation boom crossed over each stake designated. The values on the Y axis are unitless, with lower values corresponding to higher soil moisture.

**Figure 13:**
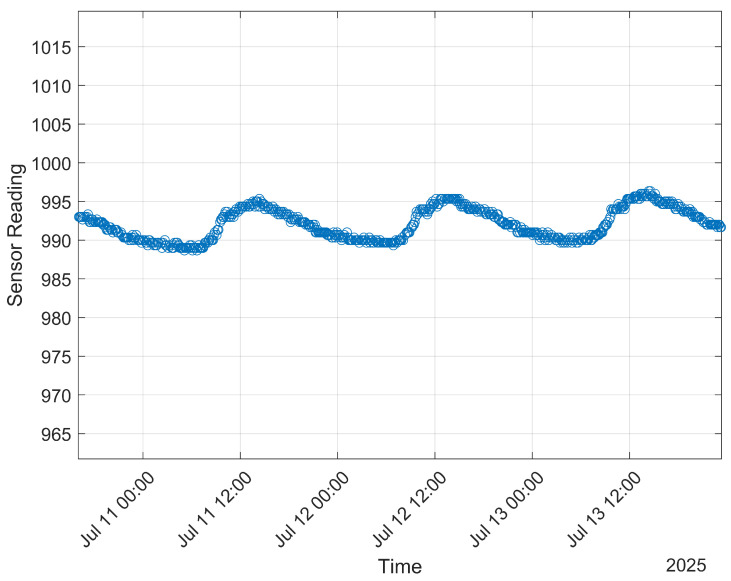
Measurements reported by Smart Bluetooth Stake 77A1 from 10 to 14 July. Y axis values are unitless, and lower values correspond with higher soil moisture and lower temperature. Note the diurnal variation.

**Figure 14:**
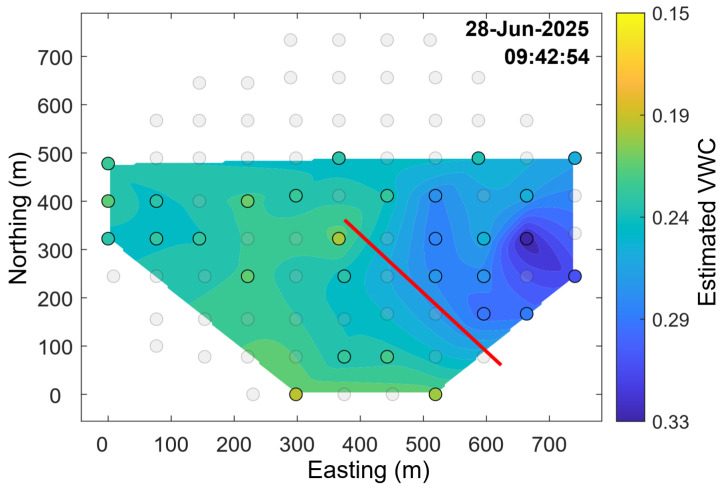
Estimated soil moisture during irrigation, with boom position (traveling clockwise) represented by the red line. Colored circles represent stakes with reported measurements at the given time with color indicating estimated VWC, and the color plot is generated with a natural neighbor interpolation. Transparent circles show the locations of stakes that did not communicate successfully during this time window. Full time series mapping over 3 days included in Supplementary Information.

A clear increase in estimated soil moisture at the time the boom crossed over the stake is evident when the raw soil moisture sensor measurements are plotted, as shown in Figure 12. Additionally, expected diurnal moisture cycles, as dew condenses onto the ground in the early morning hours and then evaporates away throughout the day, are visible in collected data for most of the sensors (see Figure 13). Data from 26 to 29 June when an irrigation sweep was in progress were used to create a time series mapping of estimated VWC, as shown in Figure 14. The entire time series mapping is included in the Supplementary Information.

### 4.4. Antenna Performance

**Figure 15:**
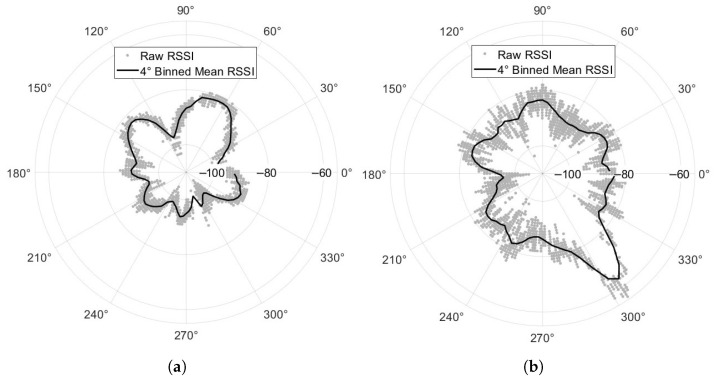
Received signal strength indicator (RSSI) vs. Smart Receiver pointing angle with respect to a single stake. RSSI data were binned every 4° and then a moving average filter with a width of 3 was applied to generate the solid line. (**a**) BGM220S board patch antenna. (**b**) Parabolic, high-gain antenna.

The successfully received packets with the parabolic antenna ranged between −48 dBm and −108 dBm, and the receive sensitivity of the BGM220S module used is −106.4 dBm with GAP-Coded PHY. The dramatic improvement from using a parabolic, high-gain antenna instead of a patch antenna is shown in Figure 15. The patch antenna has a lower baseline gain value and shows several different lobes that approach or nearly approach that baseline. The parabolic antenna shows a main lobe with about 20 dBm better RSSI than the patch antenna, as expected, and very small sidelobes. The 20 dBm increase in directivity came with a maximum reliable transmission range increase in the field from about 300 m to 600 m. However, the raw number of packets received over time stayed approximately the same between both antennas, as the patch antenna received a greater proportion of packets from nearby stakes at the cost of fewer packets from further stakes.

**Figure 16:**
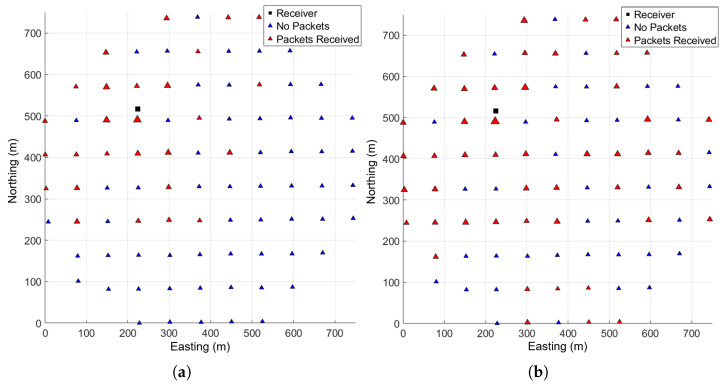
Smart Bluetooth Stakes with successful transmissions to the boom-mounted receiver (symbol size proportional to number of received packets) equipped with (**a**) patch antenna and (**b**) parabolic antenna.

After switching to the parabolic antenna, the receiver successfully received packets from nearly every part of the field, as shown in Figure 16b. The increase in packets received is particularly noticeable in the east and south corners of the field but not as much along the boom towards the southeast direction.

**Figure 17:**
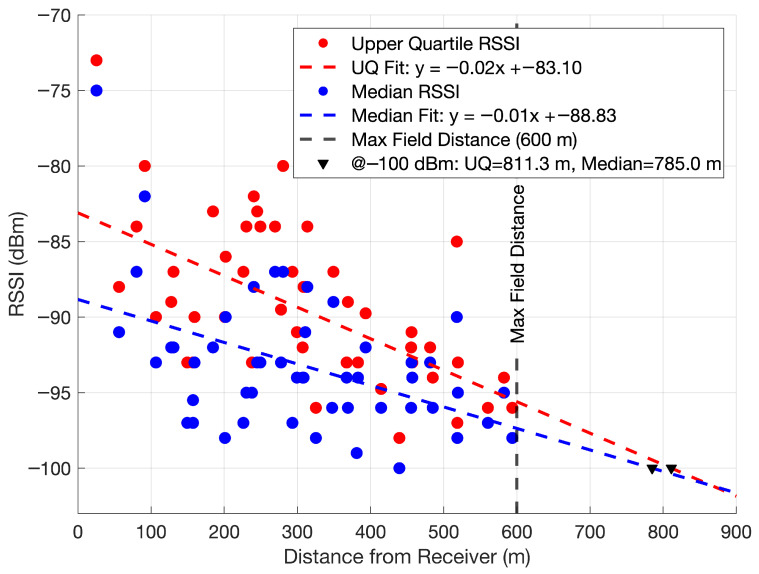
Upper quartile and median RSSI for packets obtained with the high-gain parabolic antenna receiver from 10 to 16 July versus distance to Smart Bluetooth Stakes. Least-squares fitting is used to project the estimated maximum distance beyond the 600 m field size limitation due to the static receiver position at −100 dBm.

Plotting RSSI versus distance in Figure 17 demonstrates that the maximum distance that packets could be received from stakes is beyond the edge of the field. This packet reception data was obtained with respect to the static position of the receiver with the parabolic antenna. In this plot, the upper quartile RSSI and the median RSSI values are plotted versus distance to each of the Smart Bluetooth Stakes from the receiver. For these successfully received packets, the median RSSI does not fall below about −100 dBm and the spread between the upper quartile and the median decreases as distance increases. Given the considerable receive sensitivity of the BGM220S module, a reasonable projection of about 800 m for the maximum reception distance at approximately −100 dBm is estimated.

### 4.5. Factors Impacting Reception Rates

Figure 12 and Figure 14 show that successful reception range is generally longer in front of the path of the boom than behind it, which is explained by higher signal attenuation when the wheat has been recently watered [40].

**Figure 18:**
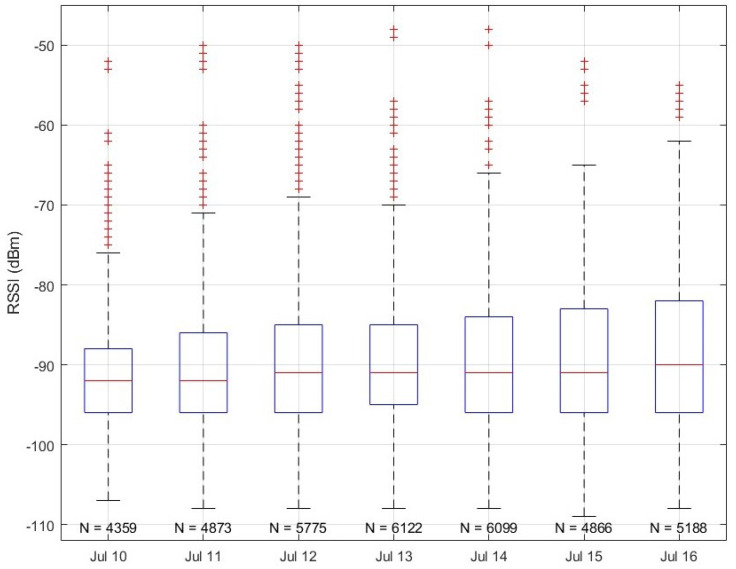
Signal strength of packets received each day after 14:00 between 10 and 16 July 2025, during which no precipitation or irrigation events occurred. Packets received before 14:00 were not counted due to a temporary dropout on the morning of 14 July. Note the steady increase in top quartile received signal strength indicator.

Because canopy water content is a significant factor affecting signal attenuation, it is expected that as the winter wheat dries out during the final pre-harvest stage, signal strengths should increase over time. This trend is visible in Figure 9, which displays a period with no precipitation events after the switch to the parabolic antenna. As the crop continued to dry, the attenuation decrease manifested as a progressive increase in the number of packets received and an increase in the signal strength of the received packets, as shown in Figure 18.

### 4.6. Boom Angle Reconstruction

GPS data from the receiver phone was less reliable than anticipated, so an effort was made to compare the phase offset between the phone magnetometer and rotator potentiometer in order to extract an estimate for the boom angle over time. Unwrapping errors in the magnetometer heading required manual correction. Once a clean magnetometer dataset was estimated, the difference between the two over time was computed and then low-pass-filtered. The resulting phase offset over time was combined with information about the start and stop times of the irrigation sweep to estimate the position of the boom throughout the sweep, as shown in Figure 14.

### 4.7. System Cost

Parts and materials to construct 105 stakes were sourced for this deployment. At that scale, the parts cost for each stake was just under USD 30 as shown in Table 1. The part cost for the boom-mounted receiver was about USD 850.

## 5. Discussion

The deployment of the Smart Bluetooth Stake solution was guided by the desire to capture data in the mature winter wheat stage to test the feasibility of the system under the most challenging transmission conditions for a given crop. The original experimental design was to additionally capture signal propagation and soil moisture profiles throughout much more of the winter wheat growing season. Challenging global conditions made sourcing and assembling materials more difficult, which reduced testing prior to field deployment.

### 5.1. Receiver Antenna Choice

The results of the field experiment show that whether a patch antenna or a parabolic dish antenna is used, the receiver system is able to consistently detect packets from sensors within the operational range of the antenna. The patch antenna is simpler, and has a reliable operating range of 150–200 m in mature winter wheat. A non-rotating patch antenna receiver could be constructed for less than half the cost of a rotating high-gain antenna receiver. For smaller fields, or larger fields where setting up multiple lower-cost receiver stations is possible and advantageous over fewer higher-cost receiver stations, the patch antenna may be the optimal choice. There is also potential for application in closed-loop irrigation management systems that are capable of using data collected from sensors in the path of the irrigation boom as it is rotating and tailoring the irrigation prescription in real time. Such a system would not be able to report the soil moisture everywhere in the field at a given time.

The parabolic high-gain antenna has significantly longer reception range, and a single receiver would likely be sufficient to survey an entire field, as long as the farmed crop had relatively low canopy density and height. Taller and denser crops like corn or potatoes could require two or more receivers for full-field continuous monitoring.

### 5.2. Canopy Attenuation

Winter wheat is a favorable crop for through-canopy transmission: it is short, it has low canopy biomass, and it does not have many surfaces on which water can condense and be held in the air in the path of transmission, as the leafy growth of other plants can. Higher Leaf Area Index (LAI) [41], greater moisture, and taller crops all present more difficult transmission environments, which will decrease the effective range of the stakes. A simple straight-line path attenuation model [42,43] for the longest successful transmission range in this experiment gives an estimate of the absorption coefficient of the winter wheat at around 0.07. In [44], the measured absorption coefficient of corn varied between 0.168 and 0.120, which according to the same simplified model could reduce the effective range of the current Smart Bluetooth Stake to as low as 80 m in 2 m tall corn. Future investigation will help to determine the maximum transmission range, and therefore the viability, of Smart Bluetooth Stakes in different crops.

### 5.3. Stake RF Matching

The antenna matching on the stakes was quite poor, with return loss of about −5 dB and standing wave ratio of 3. This means that a significant portion of the energy output from the stake transceiver was reflected back into the circuit instead of radiating out, significantly hampering transmission. The stake circuit design is based off of a reference design from Silicon Labs, which includes a specified controlled impedance layer stackup. An available layer stackup from our circuit manufacturer was thought to be close enough to the reference design that minor retuning during assembly would be sufficient. This supposition was incorrect and, due to time constraints, this problem could not be fixed for the stakes deployed in this paper. Proper tuning in future iterations will improve transmission performance by as much as several dB.

### 5.4. Measurement Accuracy

The goal for this deployment was to measure soil moisture with precision at least comparable to the ‘feel wetness’ tests discussed in [45], which about 70% of the detected stakes achieved, but the three-voltmeter method is capable of much better results with a more refined implementation. Future efforts will increase the precision and uniformity of the nail probe mounting method in order to collect more accurate data. Despite this partial success, enough data was collected that mapping of soil moisture was still achievable, demonstrating the resilience of the excess-capacity Smart Bluetooth Stake approach.

### 5.5. Solar Energy Harvesting

At time of writing, it is difficult to discern the exact cause of attrition for the 11 stakes that never successfully transmitted packets, but the 14 other stakes that were only barely able to charge enough to broadcast at 3 V suggest that insufficient power might be the problem. The simple boost–buck harvesting scheme on the current stakes is naive in that it tries to draw as much current as it can from the solar panel, which pulls the panel down to about 0.5 V in heavy shade and significantly reduces the efficiency of both the panel and the boost converter. In the current design this inefficiency was compensated for by the significant over-sizing of the solar panel relative to the average power draw of the stake, but the mixed performance of this design in the test shows the need for additional improvement. In future iterations, a dedicated energy harvesting chip with MPPT would likely be able to harvest significantly more power with the same solar panel.

## 6. Conclusions

Despite teething issues that resulted in higher than ideal stake attrition and receiver downtime, the deployment of the Smart Bluetooth Stake system in a commercial farm setting demonstrates that the architecture is capable of fulfilling its design goals. In this study, continuous direct soil moisture measurement, reliable data transmission, minimal interruption to farm activities, and cost-effectiveness have all been demonstrated. BLE is shown to be capable of reliable transmission from ground-based low-power sensors through a winter wheat canopy to boom-mounted receivers at distances well in excess of 500 m, with a maximum theoretical range greater than 800 m. In systems that naturally have low data bandwidth requirements, inherent device excess capacity, retransmission of logged data, and use of high-gain antenna configurations are important ideas that can be applied to other large wireless sensor networks.

## Figures and Tables

**Table 1:** Cost breakdown per Smart Bluetooth Stake and proportion of total cost.

Item	Cost per Stake (USD)	Proportion of Total (%)
Nail Probes	0.72	2.43
RF Shield	1.30	4.40
Solar Panel	1.60	5.43
Antenna	1.82	6.18
Enclosure	2.40	8.14
RF Power Measurement IC	2.45	8.32
Supercapacitor	2.72	9.24
MCU	3.23	10.97
PCB and Components Except MCU and RF IC	13.21	44.87
Total	29.44	100.00

## Data Availability

The data presented in this study are available on request from the corresponding author due to privacy reasons.

## Supplementary Materials

The following supporting information can be downloaded at: https://www.mdpi.com/article/10.3390/s25175537/s1, Video S1: SoilMoistureMapping.mp4

## Abbreviations

The following abbreviations are used in this manuscript:

BLE	Bluetooth Low Energy
RSSI	Received Signal Strength Indicator
EIRP	Equivalent Isotropic Radiated Power
ToA	Time Over Air
WUE	Water Use Efficiency
MPPT	Maximum Power Point Tracking
LAI	Leaf Area Index
MCU	Microcontroller Unit
WSN	Wireless Sensor Network
UUID	Universally Unique Identifier
NAIP	National Aerial Imagery Program
GPS	Global Positioning System
IMU	Inertial Measurement Unit
PCB	Printed Circuit Board
UART	Universal Asynchronous Receiver Transmitter
